# Venous thromboembolism risk and prophylaxis in the acute hospital care setting: report from the ENDORSE study in Egypt

**DOI:** 10.1186/1477-9560-10-20

**Published:** 2012-09-05

**Authors:** Hadi A Goubran, Sherif Sholkamy, Alaa El-Haddad, Alaa Mahmoud, Mounir A Rizkallah, George Sobhy

**Affiliations:** 1 Professor of Medicine Cairo University (Sabbatical), Hematologist, Saskatoon Cancer Centre, Saskatoon, Canada; 2 Professor of Vascular Surgery, Ain Shams University, Cairo, Egypt; 3 National Cancer Institutes, Cairo, Egypt; 4 Professor of Medicine, Cairo University, Cairo, Egypt; 5 Prof. of General Surgery, Ahmed Maher Teaching Hospital and Al Salam Hospital, Mohandeseen, Cairo, Egypt; 6 Shabrawishy Hospital, Cairo, Egypt

**Keywords:** Venous thromboembolism, Egypt, Thromboprophylaxis, Risk factors

## Abstract

**Background:**

Venous thromboembolism (VTE) is a leading cause of hospital-related deaths worldwide. However, the proportion of patients at risk of VTE who receive appropriate prophylaxis in Egypt is unknown. The ENDORSE study in Egypt is part of a global initiative to uncover the incidence of high-risk surgical and medical patients and determine what proportion of these patients receive appropriate VTE prophylaxis.

**Methods:**

Ten Egyptian hospitals participated in this observational study, enrolling all surgical and medical patients that met the study criteria. This resulted in a cohort of 1,008 patients in acute care facilities who underwent a retrospective chart review. Each patient’s VTE risk status and the presence or absence of appropriate prophylactic care was assessed according to the American College of Chest Physicians (ACCP) guidelines 2004.

**Results:**

Of the 1,008 patients enrolled, 395 (39.2%) were found to be at high-risk for VTE. Overall, 227 surgical patients were at high-risk, although only 80 (35.2%) received ACCP-recommended prophylaxis. Similarly, 55/268 (32.75%) of high-risk medical patients received appropriate VTE prophylaxis. Low molecular weight heparin was the most commonly used anticoagulant, while mechanical prophylactic use was quite low (1.5%) in high-risk patients.

**Conclusions:**

In Egypt, more than one-third of all patients hospitalized for surgery or acute medical conditions are at high risk for developing VTE. However, only a small fraction of these patients receive appropriate VTE prophylaxis. Corrective measures are necessary for preventing VTE morbidity and mortality in these high risk patients.

## Introduction

Venous thromboembolism (VTE) is a potential life-threatening complication that can arise during hospitalization for surgery or for medical illness. The worldwide incidence of VTE is difficult to quantify, as clinical symptoms can be non-specific and screening techniques can fail to properly assess non-symptomatic patients. Even so, it is thought that at least 5-15% of hospitalized medical patients will develop VTE, making it the most common preventable cause of in-hospital death [1,2]. The conditions that predispose patients to thromboembolism are well characterized and prophylactic measures are effective in preventing VTE complication. Immobility, endothelial injury, certain drugs, and various medical / surgical conditions (including blood coagulation disorders, malignancy, stroke, and pregnancy) can all prompt VTE development [3]. Effective prophylactic measures include treatment with anticoagulant drugs and/or mechanical devices, such as elastic graduated stockings or intermittent pneumatic compression. However, these measures can only be effective when utilized properly in patients whose VTE risk has been well-characterized.

There are a number of evidence-based guidelines for assessment of VTE risk that are recognized internationally. Assessment of VTE risk is done with Risk Assessment Model (RAM). Physicians rely mainly on ACCP guidelines, which define low, moderate, and high-risk patients based on the type of surgery, mobility status, and bleeding risk [4]. The ACCP defines high-risk patients as those undergoing hip or knee fracture surgeries, or with major trauma, and those at high VTE risk who also have a high bleeding risk. Patients that fall into the high-risk group are estimated to have between 40-80% risk of developing VTE if no prophylaxis is provided [4]. Recommended prophylaxis measures include: treatment with low molecular weight heparin (LMWH), low-dose unfractionated heparin (UFH), or fondaparinux for patients undergoing major surgery. Mechanical methods of prophylaxis are urged for patients with a high bleeding risk [4]. Unfortunately, numerous studies suggest there is a disconnect between the current knowledge on VTE prevention and implementation of appropriate prophylactic measures, since many at-risk patients do not receive prophylaxis and may not even be assessed for VTE risk [5-7].

The need to improve VTE risk assessment and prophylactic practices in the hospital setting is clear. For this reason, the ENDORSE study was conducted in 32 countries in order to ascertain the scope of the VTE risk management problem in the hospital setting [8]. The primary goals of this initiative were to [1]: examine medical and surgical patients in representative hospitals worldwide who are at risk for VTE, and [2] determine the percentage of at-risk patients who receive appropriate prophylaxis. Secondly, we sought to define the number of patients at risk of VTE by various types of acute illness and to elucidate the factors which determine prophylaxis in the acute care setting. The aim of the current publication is to present the country-specific data from the Egyptian arm of the ENDORSE study. Ten hospitals were chosen to participate, recruiting over 1,000 medical and surgical patients whose charts were analyzed for VTE risk and the presence (or absence) of prophylaxis. Our findings shed light on the scope of the global health burden associated with VTE and highlight how VTE risk is managed differently in different countries.

## Methods

### Study design

The current analysis is part of a multi-center, observational study in 32 countries known as the ENDORSE study (Epidemiological International Day for the Evaluation of Patients at Risk for Venous Thromboembolism in the Acute Hospital Care Setting) [8]. The goal of this initiative were to [1] evaluate the risk of VTE among medical and surgical patients in representative hospitals worldwide and [2] to determine the percentage of at-risk patients who receive appropriate prophylaxis. This program was developed to test the hypotheses that use of appropriate VTE prophylaxis varies by country and that prophylaxis, even when implemented, may be used suboptimally. For the Egyptian arm of the ENDORSE study, all eligible hospitals in the country were identified (n = 80). Hospitals were considered eligible for enrolment if they contained more than 50 beds, admitted patients for the treatment of medical illnesses and exacerbations of chronic diseases, and scheduled routine major surgical procedures. Non-acute and single specialty hospitals were excluded. These hospitals were chosen to be representative of the local practices and standard of care in the country. Oversampling of hospitals with known expertise in VTE was avoided. This resulted in ten participating Egyptian hospitals, which were responsible for identifying medical and surgical patients who met the study criteria. Data was collected directly from the patient medical charts by data abstractors, thus there was no influence on therapy and no patient interaction. The study data was collected retrospectively in order to capture VTE risk, prophylaxis, and bleeding risk occurring in the 14 days following patient admission. The relevant medical information was collected from eligible patients in consecutive order on the day of the study and recorded in a case report form (CRF). The survey was conducted in compliance with all appropriate local and international ethical guidelines.

### Patients

Study patients were selected in keeping with the ENDORSE initiative as previously described [8]. Briefly, medical and surgical patients were recruited for ENDORSE participation during a single hospital stay between November and December 2006 -. Surgical patients who were at least 18 years of age and who met the following criteria were included for study [1]: patient underwent surgery that both required anesthesia and lasted at least 45 minutes, and/or [2] patient had a major traumatic injury that did not require a major operation. Medical patients included in this study were over the age of 40 and were admitted for treatment of a serious acute medical illness. Patients admitted solely for diagnostic testing and those admitted to a floor not included in the study (psychiatric, maternity, etc.) were excluded from the study. In total, 1008 patients met these criteria, including 478 surgical and 530 medical patients, and were enrolled in the study.

### Reported measures

The lead investigator at each hospital recorded the following information for each study site: number of beds, approximate number of discharges per year, hospital type (public/private/academic), availability of institutional review boards, availability of wards, and the presence (or absence) of a hospital-wide DVT prevention protocol. A patient enrollment log was maintained at each hospital, which detailed relevant information about the ward/floor in question. Eligible patients were assigned a study identification number and their charts were reviewed for relevant medical information covering their first 14 days in the hospital. A CRF was used to record the patient data of interest: demographics, biometric information, medical history, date of admission, VTE risk factors, type/dose/frequency of prophylaxis, presence/absence of therapeutic anticoagulation, and discharge status. Prophylaxis was considered appropriate if it met defined recommendations of the 2004 ACCP guidelines [4]. Patients were considered to be at a significant bleeding risk if they presented with or developed intracranial hemorrhage, hepatic impairment, bleeding at admission, active gastroduodenal ulcer, or known bleeding disorder [9]. Patient and investigator data was treated in compliance with all local applicable laws and regulations. Appropriate measures were taken to safeguard data confidentiality.

### Statistical analyses

Statistical analyses are discussed fully in the global ENDORSE publication [8]. Quantitative data is presented as the median and the number of non-missing data. Categorical data is given as the number and the percentage of the population. Distribution of patients by risk factor type and by prophylaxis is provided. Use of VTE prophylaxis is presented as a function of key population characteristics. The association between VTE prophylaxis and risk factors is presented using logistic regression models and odds ratios where appropriate. In order to assess the true prevalence of VTE at 25% with a precision of ±4%, a minimum of 450 patients per analysis group was utilized.

## Results

The findings presented here are part of a larger, international, cross-sectional initiative known as the ENDORSE study [8].

**Figure 1:**
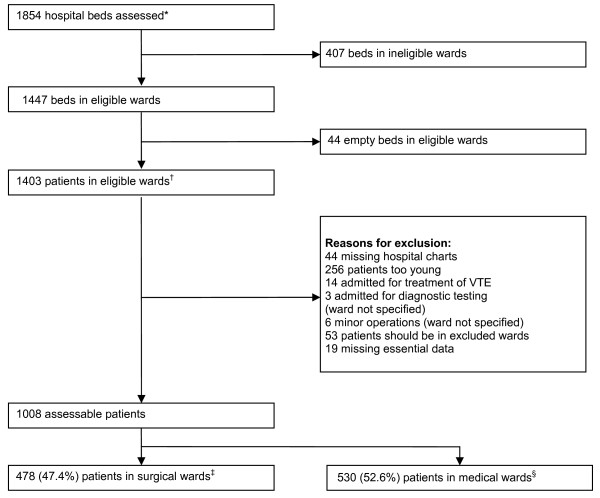
**Selection of study population and reasons for exclusion.** Abbreviations: VTE = venous thromboembolism * On basis of hospital enrolment forms ^†^ On basis of patient enrolment logs, includes patients who did not meet protocol requirements (eg., age, type of condition, or missing hospital chart) ^‡^ Includes patients in general surgical units, surgical intensive care units, neurosurgery, gynecology and orthopedics ^§^ Includes patients in other eligible wards.

**Figure 2:**
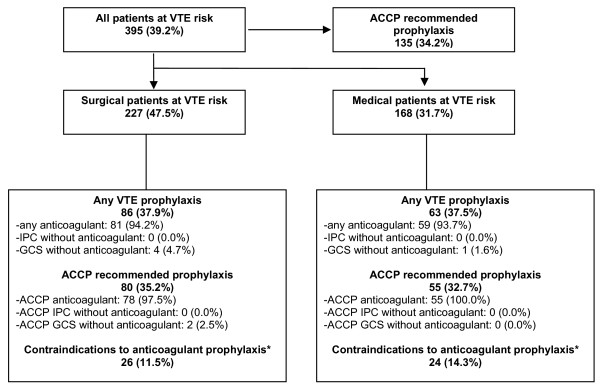
**Flowchart of high-risk patients: medical and surgical.** Abbreviations: IPC = intermittent pneumatic compression, GCS = graduated compression stockings, ACCP = American College of Chest Physicians-2004 Recommendations. * Risk factors for bleeding that were used to define "contraindications to anticoagulant prophylaxis" include any of the following conditions present at current admission: - Intracranial hemorrhage - Hepatic impairment (clinically relevant) - Bleeding at hospital admission (exclude if anticoagulant started after any type of surgery) - Active gastro-duodenal ulcer (exclude if anticoagulant started after gastric/colon/small bowel surgery) - Known bleeding disorders (congenital or acquired).

In each of the ten Egyptian hospitals that participated in ENDORSE, a log of all medical and surgical patients was generated (n = 1403), and patients were enrolled or excluded based on the criteria outlined in Figure 1. This resulted in a total of 1,008 eligible patients who were selected to undergo a chart review. In our study population, 478 (47.4%) of eligible persons were surgical patients and 530 (52.6%) were medical patients. Of the 478 surgical patients, 227 (47.5%) were found to be at high-risk of VTE, as were 168/530 (31.7%) medical patients (Figure 2). This equates to 395/1008 (39.2%) of the entire patient population meeting ACCP high-risk criteria for VTE.

### Patient characteristics and reasons for hospitalization

Characteristics of the high-risk patient population are given in Table 1. There were more high-risk men (n = 234) than women (n = 156) in our study group (60% and 40%, respectively). The average age in the high-risk surgical group was 52 years, while in the high-risk medical group it was 59 years (Table 1). The reasons for patient hospital admittance are given in Table 2. Of surgical patients with high VTE risk, the most common surgery was hepatobiliary, with 33/227 (14.5%) of patients undergoing this operation (Table 2). For the high-risk medical group, the most common reasons for admittance were cardiovascular disease (115/168, 68.5%) followed by pulmonary infection (74/168, 44%) and endocrine/metabolic disorders (43/168, 25.6%).

**Table 1:** Characteristics of high-risk patients

**Patient characteristics**	**Surgical risk**	**Medical risk**	**All risk**
	**(N = 227)**	**(N = 168)**	**(N = 395)**
Male, n/N (%)	136/225 (60.4%)	98/165 (59.4%)	234/390 (60.0%)
Age in years, median	52.0	59.0	55.0
Weight in kg, median	80.0	73.5	80.0
Height in cm, median	167.5	160.0	165.0
BMI in kg/m^2^ median	26.1	25.8	26.0
Length of hospitalization in days up to survey date, median	7.0	4.0	5.0

Abbreviations: BMI = body mass index.

Note: n is the numerator used in calculating the percentage; denominators may vary due to missing data.

**Table 2:** Reasons for hospital admission for high-risk patients

**Reasons for hospital admission**	**Surgical risk**	**Medical risk**	**All risk**
	**(N = 227)**	**(N = 168)**	**(N = 395)**
Medical Conditions			
Acute heart failure (NYHA Class III or IV)	0 (0.0%)	30 (17.9%)	30 (7.6%)
Ischemic stroke	1 (0.4%)	19 (11.3%)	20 (5.1%)
Hemorrhagic stroke	1 (0.4%)	3 (1.8%)	4 (1.0%)
Other cardiovascular disease	26 (11.5%)	63 (37.5%)	89 (22.5%)
Hematologic disease	8 (3.5%)	7 (4.2%)	15 (3.8%)
Acute non-infectious respiratory disease	3 (1.3%)	24 (14.3%)	27 (6.8%)
Pulmonary infection	13 (5.7%)	74 (44.0%)	87 (22.0%)
Malignancy (active)	66 (29.1%)	14 (8.3%)	80 (20.3%)
Infection (non-respiratory)	24 (10.6%)	12 (7.1%)	36 (9.0%)
Rheumatologic or inflammatory	0 (0.0%)	4 (2.4%)	4 (1.0%)
Neurologic	0 (0.0%)	8 (4.8%)	8 (2.0%)
Renal	10 (4.4%)	20 (11.9%)	30 (7.6%)
Endocrine/Metabolic	35 (15.4%)	43 (25.6%)	78 (19.7%)
GI/Hepatobiliary	48 (21.1%)	24 (14.3%)	72 (18.2%)
Other medical condition	23 (10.1%)	4 (2.0%)	27 (6.8%)
Type of 1^st^ Major Operation			
Hip replacement	0 (0.0%)	N/A	N/A
Knee replacement	2 (0.9%)	N/A	N/A
Hip fracture	6 (2.6%)	N/A	N/A
Curative arthroscopy	1 (0.4%)	N/A	N/A
Other ortho trauma	23 (10.1%)	N/A	N/A
Colon/Small bowel	19 (8.4%)	N/A	N/A
Rectosigmoid	4 (1.8%)	N/A	N/A
Gastric	8 (3.5%)	N/A	N/A
Hepatobiliary	33 (14.5%)	N/A	N/A
Urologic	19 (8.4%)	N/A	N/A
Vascular	11 (4.8%)	N/A	N/A
Thoracic	4 (1.8%)	N/A	N/A
Gynecologic	10 (4.4%)	N/A	N/A
Other major surgery	78 (34.4%)	N/A	N/A
Admitted with major trauma but surgery not performed	9 (4.0%)	N/A	N/A

Abbreviations: NYHA = New York Heart Association, GI = gastrointestinal.

Note: n is the numerator used in calculating the percentage; denominators may vary due to missing data.

### VTE risk factors in patient population

The VTE risk factors present before and during hospitalization are provided in Table 3. Many high-risk patients had conditions that put them at risk for VTE prior to admission, such as chronic pulmonary disease (54/395, 13.9%), obesity (25/395, 6.4%) and chronic heart failure (23/395, 5.9%). Pulmonary infection and malignancy were also common in these patients during their hospitalization (Table 3). Admission to ICU/CCU occurred in 108/395 (27.3%) high-risk patients (Table 3). Additionally, a significant proportion (75/395, 19.0%) of high-risk patients underwent complete immobilization, and another 44 (11.1%) were immobile with bathroom privileges.

**Table 3:** VTE risk factors prior to and during patient hospitalization

**VTE risk factors**	**Surgical risk**	**Medical risk**	**All risk**
	**(N = 227)**	**(N = 168)**	**(N = 395)**
Conditions Present Prior to Hospital Admission			
Previous venous thromboembolism	6 (2.7%)	1 (0.6%)	7 (1.8%)
Thrombophilia (laboratory documented)	0 (0.0%)	0 (0.0%)	0 (0.0%)
Varicose veins or venous insufficiency	13 (5.9%)	5 (3.0%)	18 (4.6%)
Post-menopausal hormone replacement therapy	2 (0.9%)	0 (0.0%)	2 (0.5%)
Chronic pulmonary disease	22 (10.0%)	32 (19.0%)	54 (13.9%)
Long term immobility	5 (2.3%)	5 (3.0%)	10 (2.6%)
Pregnancy (within 3 months)	1 (0.5%)	0 (0.0%)	1 (0.3%)
Obese (based on physician's note)	16 (7.3%)	9 (5.4%)	25 (6.4%)
Contraceptives	2 (0.9%)	1 (0.6%)	3 (0.8%)
Chronic heart failure	0 (0.0%)	23 (13.7%)	23 (5.9%)
Risk Factors for VTE During Hospital Admission			
Admitted to ICU/CCU	37 (16.3%)	71 (42.3%)	108 (27.3%)
Central venous catheter	22 (9.7%)	14 (8.3%)	36 (9.1%)
Mechanical ventilation	4 (1.8%)	9 (5.4%)	13 (3.3%)
Immobile with bathroom privileges	19 (8.4%)	25 (14.9%)	44 (11.1%)
Complete immobilization	36 (15.9%)	39 (23.2%)	75 (19.0%)
Cancer therapy	2 (0.9%)	9 (5.4%)	11 (2.8%)
Heparin induced thrombocytopenia	0 (0.0%)	0 (0.0%)	0 (0.0%)

Abbreviations: VTE = venous thromboembolism, ICU = intensive care unit, CCU = cardiac care unit.

Note: n is the numerator used in calculating the percentage; denominators may vary due to missing data.

Of the 395 patients at high-risk for VTE, 50 (12.7%) had risk factors for bleeding and thus were considered to have a contraindication to pharmacological prophylaxis. The most common bleeding risk factors differed between the surgical and medical patient groups (Table 4). For example, surgical patients were more likely to have taken non-aspirin non-steroidal anti-inflammatory drugs upon admission (10.1%), while medical patients were more likely to have taken aspirin (20.8%) or exhibited significant renal impairment (13.1%).

**Table 4:** Incidence of bleeding risk and contraindications to anticoagulant therapies

**Risk factors for bleeding present at current admission**	**Surgical risk**	**Medical risk**	**All risk**
	**(N = 227)**	**(N = 168)**	**(N = 395)**
Significant renal impairment	6 (2.6%)	22 (13.1%)	28 (7.1%)
Intracranial hemorrhage	0 (0.0%)	1 (0.6%)	1 (0.3%)
Low platelet count (<100,000 per μl)	6 (2.6%)	10 (6.0%)	16 (4.1%)
Known bleeding disorder (congenital or acquired)	1 (0.4%)	3 (1.8%)	4 (1.0%)
Hepatic impairment (clinically relevant)	7 (3.1%)	16 (9.5%)	23 (5.8%)
Bleeding at hospital admission	19 (8.4%)	3 (1.8%)	22 (5.6%)
Active gastroduodenal ulcer	2 (0.9%)	4 (2.4%)	6 (1.5%)
Aspirin on admission	12 (5.3%)	35 (20.8%)	47 (11.9%)
NSAID on admission (excluding aspirin)	23 (10.1%)	10 (6.0%)	33 (8.4%)

Abbreviations: NSAID = non-steroidal anti-inflammatory drug.

Note: n is the numerator used in calculating the percentage; denominators may vary due to missing data.

### Proportions and types of VTE prophylaxis

We found that 39.2% (395/1008) of all patients included in the study were considered to be at high risk of VTE. Of these, only 34.2% (135/395) received ACCP-recommended prophylaxis. Of the 227 surgical patients with VTE risk, 80 (35.2%) received ACCP-recommended prophylaxis, although a slightly higher proportion, 86/227 (37.9%), received any type of prophylaxis (Figure 2). Similarly, 63/268 (37.5%) of high-risk medical patients received any kind of prophylaxis, with 55 (32.7%) receiving anticoagulant prophylaxis that met ACCP recommendations. A small percentage (11.5% of surgical and 14.3% of medical patients) had contraindications to anticoagulant prophylaxis (Figure 2).

Interestingly, the proportion of patients receiving prophylaxis appears to depend on their reason for admittance (Table 5). For example 100% of surgical patients presenting with hip fractures or for rectosigmoid surgery were given thromboprophylaxis, while only 21.2% of patients undergoing hepatobiliary surgery received any prophylactic measure. Similar variability is seen among the medical patients, although no group reached 100% prophylaxis (Table 5). The medical patients most likely to undergo prophylaxis were ischemic stroke patients, with 63.2% receiving ACCP-recommended prophylaxis, respectively. In contrast, only 16.7% of acute non-infectious respiratory disease patients received VTE prophylaxis of any kind.

**Table 5:** Rates and types of VTE prophylaxis based on diagnosis

**Type of diagnosis**	**Any Px received N**	**Any anticoagula**	**GCS w/o anticoagulant/IPC**	**ACCP Px received**	**ACCP anticoagulant**	**ACCP GCS w/o anticoagulant/IPC**
	**(%)**	ntn (%)*	n (%)*	**N (%)**	n (%)*	n (%)*
**Surgical Patients at VTE Risk**						
Hip replacement	0 (0.0%)	0 (0.0%)	0 (0.0%)	0 (0.0%)	0 (0.0%)	0 (0.0%)
Knee replacement	1 (50.0%)	1 (100.0%)	0 (0.0%)	0 (0.0%)	0 (0.0%)	0 (0.0%)
Hip fracture	6 (100.0%)	6 (100.0%)	0 (0.0%)	6 (100.0%)	6 (100.0%)	0 (0.0%)
Curative arthroscopy	0 (0.0%)	0 (0.0%)	0 (0.0%)	0 (0.0%)	0 (0.0%)	0 (0.0%)
Other ortho trauma	13 (56.5%)	13 (100.0%)	0 (0.0%)	11 (47.8%)	11 (100.0%)	0 (0.0%)
Colon/small bowel	8 (42.1%)	8 (100.0%)	0 (0.0%)	8 (42.1%)	8 (100.0%)	0 (0.0%)
Rectosigmoid	4 (100.0%)	3 (75.0%)	1 (25.0%)	3 (75.0%)	3 (100.0%)	0 (0.0%)
Gastric	4 (50.0%)	4 (100.0%)	0 (0.0%)	4 (50.0%)	4 (100.0%)	0 (0.0%)
Hepatobiliary	7 (21.2%)	6 (85.7%)	1 (14.3%)	7 (21.2%)	6 (85.7%)	1 (14.3%)
Urologic	5 (26.3%)	5 (100.0%)	0 (0.0%)	5 (26.3%)	5 (100.0%)	0 (0.0%)
Vascular	6 (54.5%)	5 (83.3%)	1 (16.7%)	6 (54.5%)	5 (83.3%)	1 (16.7%)
Thoracic	2 (50.0%)	2 (100.0%)	0 (0.0%)	2 (50.0%)	2 (100.0%)	0 (0.0%)
Gynecologic	5 (50.0%)	5 (100.0%)	0 (0.0%)	5 (50.0%)	5 (100.0%)	0 (0.0%)
Other surgery	22 (28.2%)	20 (90.9%)	1 (4.5%)	20 (25.6%)	20 (100.0%)	0 (0.0%)
Major trauma but surgery not performed	3 (33.3%)	3 (100.0%)	0 (0.0%)	3 (33.3%)	3 (100.0%)	0 (0.0%)
**Medical Patients at VTE Risk**						
Acute heart failure (NYHA Class III or IV)	12 (40.0%)	11 (91.7%)	0 (0.0%)	9 (30.0%)	9 (100.0%)	0 (0.0%)
Ischemic stroke	12 (63.2%)	12 (100.0%)	0 (0.0%)	12 (63.2%)	12 (100.0%)	0 (0.0%)
Hemorrhagic stroke	2 (66.7%)	2 (100.0%)	0 (0.0%)	2 (66.7%)	2 (100.0%)	0 (0.0%)
Other cardiovascular disease	36 (57.1%)	34 (94.4%)	0 (0.0%)	32 (50.8%)	32 (100.0%)	0 (0.0%)
Hematologic disease	0 (0.0%)	0 (0.0%)	0 (0.0%)	0 (0.0%)	0 (0.0%)	0 (0.0%)
Malignancy (active)	0 (0.0%)	0 (0.0%)	0 (0.0%)	0 (0.0%)	0 (0.0%)	0 (0.0%)
Acute non-infectious respiratory disease	4 (16.7%)	3 (75.0%)	1 (25.0%)	3 (12.5%)	3 (100.0%)	0 (0.0%)
Pulmonary infection	22 (29.7%)	20 (90.9%)	0 (0.0%)	20 (27.0%)	20 (100.0%)	0 (0.0%)
Infection (non-respiratory)	3 (25.0%)	3 (100.0%)	0 (0.0%)	2 (16.7%)	2 (100.0%)	0 (0.0%)
Rheumatotologic/Inflammatory	1 (25.0%)	1 (100.0%)	0 (0.0%)	1 (25.0%)	1 (100.0%)	0 (0.0%)
Neurologic	3 (37.5%)	3 (100.0%)	0 (0.0%)	3 (37.5%)	3 (100.0%)	0 (0.0%)
Renal	7 (35.0%)	7 (100.0%)	0 (0.0%)	7 (35.0%)	7 (100.0%)	0 (0.0%)
Endocrine/Metabolic	19 (44.2%)	17 (89.5%)	0 (0.0%)	17 (39.5%)	17 (100.0%)	0 (0.0%)
GI/Hepatobiliary	5 (20.8%)	5 (100.0%)	0 (0.0%)	5 (20.8%)	5 (100.0%)	0 (0.0%)
Other medical condition	2 (50.0%)	2 (100.0%)	0 (0.0%)	2 (50.0%)	2 (100.0%)	0 (0.0%)

Abbreviations: VTE = venous thromboembolism, Px = prophylaxis, IPC = intermittent pneumatic compression, GCS = graduted compression stockings, w/o = without, ACCP = American College of Chest Physicians, NYHA = New York Heart Association, GI = gastrointestinal* The denominator is patients who received prophylaxis

IPC without Anticoagulation n = 0.

ACCP IPC w/o Anticoagulant n = 0.

The most common type of prophylaxis administered was treatment with an ACCP-recommended anticoagulant, which was given to 78 surgical and 55 medical patients (Table 5). Of these, LMWH was most commonly prescribed, with 32.6% of high-risk surgical and 28.6% of high-risk medical patients receiving this form of prophylaxis. Use of UFH, vitamin K-agonists, and other anticoagulants were much less common (Table 6). Very few at-risk patients were given mechanical prophylaxis. Graduated compression stockings (GCS) were used for only one medical and five surgical patients. No high-risk patients received intermittent pneumatic compression (IPC) or foot pump treatments in the absence of anticoagulant therapy.

**Table 6:** An overview of VTE prophylaxis ordered during hospitalization

**Type of VTE prophylaxis**	**Surgical risk**	**Medical risk**	**All risk**
	**(N = 227)**	**(N = 168)**	**(N = 395)**
Anticoagulant prophylaxis			
Low molecular weight heparin	74 (32.6%)	48 (28.6%)	122 (30.9%)
Unfractionated heparin	6 (2.6%)	7 (4.2%)	13 (3.3%)
Vitamin-K antagonist	8 (3.5%)	9 (5.4%)	17 (4.3%)
Fondaparinux	0 (0.0%)	0 (0.0%)	0 (0.0%)
Other anticoagulants	1 (0.4%)	3 (1.8%)	4 (1.0%)
Mechanical prophylaxis			
Intermittent pneumatic compression	0 (0.0%)	0 (0.0%)	0 (0.0%)
Foot pump	0 (0.0%)	0 (0.0%)	0 (0.0%)
Graduated compression stockings	5 (2.2%)	1 (0.6%)	6 (1.5%)

Abbreviations: VTE = venous thromboembolism.

Note: n is the numerator used in calculating the percentage; denominators may vary due to missing data.

## Discussion

Egyptian patients enrolled in Endorse exhibit nearly the same features as compared to the global study. First, in both the global and Egyptian studies, surgical patients make up a slightly higher percentage of high-risk patients than do medical patients. Second, risk factors for bleeding present at the time of admission were similar in both studies, with aspirin consumption being the most common in both cohorts. Third, the Egyptian cohort exhibited many trends in prophylactic usage that were seen in the global report. In both global and Egyptian patient groups, the majority of those given prophylaxis received thromboprophylaxis that met ACCP guidelines. On both the international and Egypt-specific levels, there was substantial room for improvement to achieve a goal of 100% prophylaxis compliance, as only ~30-50% of high-risk patients received any preventative care. The most common class of anticoagulant given to patients globally and Egyptian patients specifically, were LMWH to ~30%-40%. Finally, in both studies, surgical patients were more likely to receive heparin prophylaxis than medical patients. This is consistent with other research, which found that VTE prophylaxis is less often provided to medical patients than surgical patients, despite a similar risk [10].

There were however some important differences between the Egyptian and global patient population analyzed in this initiative. In the global study, 51.8% of all patients were found to be at high-risk for VTE, compared to only 39.2% of Egyptian patients. One reason that the Egyptian arm of this study identified fewer at-risk patients than the global cohort may be related to the demographics of the Egyptian group. In the Egyptian study, there was a 60% to 40% split of men to women, whereas the genders were more equally represented in the global cohort. The Egyptian study population was also ten years younger, on average, than the global patient group (55 years vs. 65 years of age, respectively). This may have had a significant impact on the overall VTE risk rate, as incidence of VTE has been shown to increase with age in numerous studies [3]. Additionally, the Egyptian group exhibited lower incidences of obesity and of various other medical conditions (such as chronic heart failure, pulmonary infection, and rheumatologic, inflammatory, neurologic, and metabolic diseases) than the global cohort. Finally, Egyptian patients seemed to have fewer bleeding risk factors overall during their hospital stay, with much lower rates of central venous catheterization and mechanical ventilation. The combination of all of these factors may account for the lower risk of VTE in the Egyptian cohort.

Although the Egyptian cohort had a lower overall rate of high-risk patients, we found that a much lower percentage of such at-risk cases received appropriate prophylaxis compared to the global survey. In the Egyptian hospitals sampled, only 34.2% of those at high VTE risk received ACCP-recommended prophylaxis, while globally, appropriate prophylaxis was given to 50.2%. The rate of prophylactic usage in Egypt is comparable to the rates in Greece, Pakistan, and the United Arab Emirates, ranging between the lowest (3%, Bangladesh) and highest (70%, Germany) rates of prophylaxis globally [8]. Of course, there still remains great room for improvement in all instances, with the potential for enormous benefits if 100% compliance could be achieved. The reasons behind such modest rates of VTE prophylaxis are likely to be numerous and highly dependent on the specifics of medical practices in various countries; thus further examination on a hospital-specific level is needed.

While surgical patients were more likely to receive prophylaxis in the global study, near equal rates of prophylactic use was observed for both the Egyptian surgical and medical groups. This equality in prophylactic use between the two groups may be due to the fact that prophylactic utilization was low overall in Egypt, regardless of what type of medical condition/procedure was being addressed. Those undergoing most types of surgery (including knee-replacements, bowel, gastric, hepatobiliary, urologic, vascular, thoracic, and gynecological) had prophylaxis rates substantially lower than in the global study. A similar trend was seen in the majority of Egyptian medical patients, with low rates of prophylaxis independent of the reason for hospitalization. We also recorded very low rates of mechanical prophylaxis in Egypt, with no high-risk patients receiving IPC or AVI and only 1.5% receiving GCS. This finding was not surprising, as other international studies also have reported low rates of IPC mechanical prophylaxis outside of the United States [11].

This study had a number of limitations that are important to consider. Mainly, the findings are based on patients receiving care in 10 hospitals, which represents ~12.5% of all of the hospitals in Egypt. While the country-wide rates of VTE prophylaxis may differ, the low number of high-risk patients whose VTE needs are being addressed in this study suggests there is an immediate need to improve VTE risk management in Egypt. Additionally, the study data only represents the first 14 days of medical care after admission, thus our findings may underestimate the true incidence of VTE.

Literature comparing rates of potentially-life threatening but preventable complications of hospitalization between Egypt and other areas of the world is scarce. Thus another limitation of our study is the lack of comparable data to aid in the interpretation of these observed trends. A recent study comparing coronary surgery in Egypt and Germany found that Egyptian patients had much higher incidence of hospital-related mortality compared to their western counterparts (6.1% vs. 1.7%, respectively) [12]. Another study of Egyptian patients with cerebral venous thrombosis revealed that only 67% received therapeutic anticoagulant treatment, compared to 85% of patients in an international cohort [13]. That study also found higher rates of morbidity and mortality after the thrombotic event in Egypt compared to other countries (51% vs. 19%), though the authors suggest this may be attributable, in part, to more severe disease at presentation [13].

Thus, we have reason to believe that our findings are consistent with the trends seen in other hospital-based surveys of Egyptian patient care.

As a leading cause of hospital-related, preventable morbidity and mortality, VTE contributes significantly to health burden around the world. The data presented here reveal that, like many other countries surveyed, VTE risk assessment and prophylaxis is underutilized in the Egyptian acute care setting. In order to raise the rates of appropriate prophylaxis towards a goal of 100% implementation in an effective manner, we must understand why so many high-risk patients fail to get the prophylactic measures they need. Education aimed at increasing VTE awareness in medical care providers, as well as implementation of VTE risk assessment and prophylaxis protocols in every hospital, is immediately necessary to address this widespread problem.

## Competing interests

The authors declare that they have no competing interests.

## Authors’ contributions

HG, SS, AH, AM, and GS, participated in reviewing the patients records and collected the relevant data. HG and SS drafted the manuscripts. All authors have read and approved the final manuscript.
